# Deciphering aroma formation during flowering in nectar tree (*Tilia amurensis*): insights from integrated metabolome and transcriptome analysis

**DOI:** 10.48130/FR-2023-0024

**Published:** 2023-10-08

**Authors:** Kewei Cai, Qiushuang Zhao, Hanxi Li, Qinhui Zhang, Yan Li, Rui Han, Tingbo Jiang, Xiaona Pei, Lina Zhang, Xiyang Zhao

**Affiliations:** 1 Jilin Provincial Key Laboratory of Tree and Grass Genetics and Breeding, College of Forestry and Grassland Science, Jilin Agricultural University, Changchun, China; 2 State Key Laboratory of Tree Genetics and Breeding, Northeast Forestry University, Harbin, China; 3 School of information technology, Jilin Agricultural University, Changchun, China

**Keywords:** *Tilia amurensis*, Aroma formation, Terpenoid biosynthesis, Phenylpropanoid biosynthesis

## Abstract

*Tilia amurensis* is a significant ornamental and economically-important tree species, known for its fragrant flowers, which are a source of high-quality honey production. However, the regulatory mechanisms involved in aroma formation during flower development in *T. amurensis* remains limited. The current study revealed the detection of plant hormones at every assessed stage of flower development. Among them, auxin and brassinosteroid contents significantly increased at stage 3, potentially regulating crucial functions during *T. amurensis* flower development. Moreover, the study examined the levels and change patterns of secondary metabolites and employed a combination of transcriptomics and metabolomics to comprehensively assess essential structural genes implicated in the biosynthesis pathways of terpenoid and phenylpropanoid. A comprehensive set of 89,526 differentially expressed genes (DEGs) was uncovered, including candidate structural genes *ACAT*, *HDS*, *TPS*, *4CL*, *CAD*, and *CCOAMT,* which are specifically involved in the biosynthesis of terpenoids and phenylpropanoids. Maslinic acid, 2α,3α-dihydroxyursolic acid, and betulinic acid were accumulated in the terpenoid biosynthesis pathway. In contrast, metabolites with differential accumulation, such as phenylalanine, coniferyl alcohol, and cinnamic acid, were specifically enriched in the phenylpropanoid biosynthesis pathway. The *C2H2*, *MYB*, and *NAC* transcription factor families are crucially associated with the terpenoid and phenylpropanoid biosynthesis pathways. Two transcription factors, *C2H2-17* and *MYB-24*, exhibited strong co-expression with structural genes in two networks, and were identified as central regulatory factors. These findings establish a solid groundwork for elucidating the generation of floral fragrance and provide comprehensive genetic and metabolic information for further studies on *T. amurensis*.

## Introduction

*Tilia amurensis* is among the most valuable species of the Tiliaceae family, with an expansive range of uses^[1]^. Currently, it has been classified as a second-level protected plant in China. Industrial oils can be extracted from the seeds of *T. amurensis*, and its wood can be used to make building materials. *T. amurensis* possesses an elegant tree structure, exhibits golden autumn foliage, and showcases remarkable ornamental characteristics, making it an ideal choice as a street tree species^[2]^. Additionally, *T. amurensis* flowers can release odorous fragrances and are an excellent nectar source. *Tilia amurensis* can also be used as medicine and is a source of traditional Chinese medicine for clearing heat and relieving skin^[3]^. Noteworthily, *T. amurensis* has been favored since ancient times because of its characteristic aromatic flavors, and its flower aroma quality has a major impact on the flavors of linden honey. In general, immature flower buds of *T. amurensis* are odorless, and only mature flowers give off their characteristic aroma during flowering, which lasts for about a week^[4]^. Moreover, aromatic flavors play crucial roles in maintaining the ecological associations between flowers and pollinators^[5,6]^. The synthesis of aromatic compounds in flowers is a complex process that encompasses secondary metabolism. The synthesis and degradation of compounds give rise to intricate networks of metabolites and regulatory mechanisms, involving diverse pathways^[7]^. Presently, the application of various metabolite detection platforms has greatly accelerated the exploration of plant secondary metabolite biosynthesis pathways^[8]^. Despite previous investigations into the chemical composition of *T. amurensis* flowers^[9]^, it is imperative to assess the secondary metabolite profile throughout different stages of flower development. This is particularly significant given the rising importance of *T. amurensis* in the food industry.

In recent years, the exploration of plant development has revealed the multifaceted roles of plant hormones in orchestrating the intricate process of flowering. Among the array of hormonal cues, gibberellins, brassinosteroids, auxins, cytokinins, and ethylene have emerged as central players in regulating the initiation and progression of floral development^[10]^. Gibberellins, known for their influence on stem elongation, have also been implicated in promoting floral meristem development, thus influencing the timing and dimensions of blooming^[11]^. Auxins, in their diverse functions, contribute to the positioning and differentiation of floral organs, sculpting the delicate architecture of blossoms^[12]^. Cytokinins, integral to cell division processes, intricately shape the growth of floral structures, further underscoring their significance in flowering patterns^[13]^. Ethylene, a gaseous hormone, governs not only the acceleration of flower senescence but also influences the emission of alluring floral fragrances^[14]^. Furthermore, by influencing biochemical processes such as cell elongation, differentiation, and division, brassinolide also participates in regulating the formation and development of floral organs^[15]^. Existing studies have indicated that gibberellins, brassinolide, and ethylene signaling were involved in flower differentiation and development in *Nelumbo nucifera*^[16]^. In the ornamental plant *Hedychium coronarium*, research has revealed that under the influence of auxin induction, transcription factors *HcMYB1* and *HcMYB2* are specifically expressed in flowers, regulating the biosynthesis of floral scent compounds^[17]^. Additionally, Janowska et al.^[11]^ discovered that cytokinins and gibberellins can promote the flowering and post-harvest longevity of the colorful inflorescence spathes of Calla Lilie. In conclusion, plant hormones play a significant role in flower development, influencing not only the formation and growth of floral organs but also potentially affecting the generation of floral fragrances.

The intricate biochemical pathways associated with the synthesis of floral aroma compounds mainly include pathways of terpenoids, phenylpropanoids, and fatty acid-derived volatile biosynthesis^[18]^. So far, terpenoids, a diverse family of secondary metabolites, have been considered the main aroma components of flowers and have been well-studied in plants. The possible terpenoid biosynthesis pathways have been identified in plants. Monoterpenes (C10) and sesquiterpenes (C15) constitute the major components of terpenoids. Isopentenyl pyrophosphate (IPP) and dimethylallyl diphosphate (DMAPP) serve as the primary C5 carbon precursors for the synthesis of these compounds. The mevalonic acid (MVA) pathway and the plastidial 2-C-methylerythritol 4-phosphate (MEP) pathway serve as distinct biosynthetic routes for the synthesis of these compounds, providing the precursors required for their formation^[19]^. IPP is derived from acetyl-CoA, whereas the MVA pathway is responsible for producing precursors for sterols, and sesquiterpenes. The MEP pathway utilizes pyruvate and glyceraldehyde-3-phosphate (G3P) to synthesize IPP and DMAPP, which subsequently contribute to the production of monoterpenes, and diterpenes^[20,21]^. The relationship between terpenoid biosynthesis regulatory genes and aroma production has been established in different plants, such as cucumber^[22]^, jasmine^[23]^, and celery^[24]^. Despite terpenoids being the primary constituents of volatile compounds, the genes responsible for terpenoid biosynthesis in *T. amurensis* flowers remain unidentified.

Benzenoids, especially phenylpropanoids, are metabolites with key functions in plants and are the main components of floral aroma, contributing to the attraction of pollinators by plants^[25]^. Among the various secondary metabolite pathways in plants, the benzenoid pathway has garnered significant attention and has been extensively studied. Benzenoids and phenylpropanoids (BPs) are synthesized from phenylalanine (Phe) and tyrosine, two aromatic amino acids in the shikimic acid pathway^[26]^. Phenylpropanoid metabolites contain an aromatic ring that is linked to a 3-C propane side chain^[27]^. The incorporation of diverse aromatic ring substituents and the positioning of the propylene double bond give rise to an extensive array of bioactive compounds with remarkable variations^[28]^. The biosynthesis of phenylpropanoid metabolites, including cinnamic acid, involves a coordinated cascade of enzymatic reactions. The enzymatic modification of the core structure of these metabolites relies on key enzymes such as cinnamate-4 hydroxylase (C4H), cinnamoyl-CoA reductase (CCR), and 4-coumarate-CoA ligase (4CL), which play crucial roles in this process^[27,29]^. Many studies have revealed that aroma production is related to sophisticated mechanisms that regulate the biosynthesis of phenylpropanoid metabolites, such as those reported in tea^[26]^ and tomatoes^[30]^.

The regulation of gene expression in terpenoid and phenylpropanoid biosynthesis is significantly influenced by the involvement of transcription factors (TFs), which play crucial roles in these processes^[5,21]^. Numerous TFs examined thus far have been implicated in the biosynthesis of terpenoids. Within *Catharanthus roseus*, the *AP2/ERF* transcription factor known as *ORCA3* governs the biosynthesis of terpenoid indole alkaloids^[31]^. In *Cymbidium faberi*, *MYC* TFs were identified as candidates participating in the biosynthesis of flower scents^[32]^. Furthermore, *R2R3-MYB* genes has been discovered to exert a significant regulatory function in terpenoid biosynthesis, particularly within subgroups 4, 5, and 15^[33,34]^. Several transcriptional regulators have been identified that modulate phenylpropanoid, flavonoid, and benzenoid accumulation. In petunia, *PhMYB4* regulates floral aroma by repressing the transcription of the cinnamate-4-hydroxylase gene, shifting the metabolic flux toward the synthesis of volatile phenylpropanoids^[35]^. Moreover, *ODO1*, an *R2R3-MYB* TF, has emerged as the pioneering TFs identified to regulate floral aroma production. It exerts its regulatory influence by modulating the expression pattern of various genes related to the phenylpropanoid pathway^[36]^. Despite numerous studies have investigated the regulatory role of TFs in the biosynthesis pathways of terpenoids and phenylpropanoids, the specific gene regulatory mechanisms in *T. amurensis* are still not fully understood.

In the current study, fresh flowers at different growing stages of *T. amurensis* were collected. We aimed to investigate the changes in key secondary metabolites of *T. amurensis* during flowering and elucidate the roles and involvement of key genes in the synthesis of key metabolites. We profiled the flower metabolites during different flowering stages by metabolomics to achieve these aims. Subsequently, transcriptome sequencing was conducted to discover crucial structural genes and TFs responsible for regulating the biosynthesis of terpenoids and phenylpropanoids. The phytohormone content was determined during flower development, and the expression profiles of DEGs related to phytohormone biosynthesis and signal transduction were examined. Additionally, the reliability of the transcriptome data was substantiated through RT-qPCR (real time quantitative polymerase chain reaction) analysis. This investigation will provide valuable perspectives on the molecular mechanisms governing terpenoid and phenylpropanoid biosynthesis, thus establishing a basis for targeted regulation of crucial metabolic elements within flowers of *T. amurensis*.

## Materials and methods

### Plant samples

The adult *T. amurensis* plants were cultivated at Northeast Forestry University in Harbin, China (coordinates: 126°37'57.28" E, 45°43'6.53" N). Flower samples at three distinct stages of flower development (S1, S2, and S3, respectively) were collected. The fresh flower samples from the S1, S2, and S3 stages were collected on June 5, 2022, June 15, 2022, and June 25, 2022, respectively. To minimize the impact of circadian rhythms on the results, each sampling was consistently conducted between 9 am and 10 am. A total of 5 g of each sample was collected for subsequent analysis, with three biological replicates obtained for each sample. Following collection, all samples were promptly placed in liquid nitrogen and preserved at a temperature of −80 °C for subsequent analyses. These samples were used for hormone concentration measurements, transcriptome sequencing (RNA-seq), and metabolite extraction.

### Phytohormone concentration measurements

At various stages of flower development, the concentrations of cytokinin (CTK), auxin (IAA), abscisic acid (ABA), ethylene (ETH), gibberellin (GA), zeatin (ZT), a type of cytokinin, brassinosteroids (BR), and jasmonic acid (JA) were quantified. The phytohormone content in each flowering stage was analyzed in three biological replicates. Each replicate involved the collection of 50 mg of fresh sample, and subsequent analysis of phytohormone concentrations in the extracts was performed using the high-performance liquid chromatography-mass spectrometry (HPLC-MS) and enzyme-linked immunosorbent assay (ELISA) methods^[37−39]^. The statistical analysis of the data was carried out using IBM Crop's SPSS 26.0 software (Armonk, NY, USA) in addition to Microsoft Office Excel software. To evaluate the statistical significance of variations among different samples, the Student–Newman–Keuls (S–N–K) test was employed, with a significance threshold set at *p* < 0.05.

### Sampling preparation and metabolite analysis in flowers

Wuhan Metware Biotechnology Co., Ltd. employed ultra-high performance liquid chromatography-mass spectrometry (UPLC-MS) and gas chromatography mass spectrometry (GC/MS) for extraction, and quantification of metabolites in the samples. In a concise manner, each freeze-dried flower sample underwent individual grinding into powder under liquid nitrogen in three biological replicates for every flowering stage. For LC-MS, 3 mL of the sample was used after mixing. It was placed in a 50 mL centrifuge tube, and then the intact sample was immersed in liquid nitrogen. Then the sample was placed into a lyophilizer for freeze-drying. After lyophilization, 100 uL of 70% methanol internal standard extract was added. The samples were centrifuged (21,560 × g, 4 °C) for 3 min. The resulting supernatant was passed through a micropore filter membrane, subsequently stored in a flask. The LC-MS system (UPLC, SHIMADZU Nexera X2) was utilized for the analysis of the sample extracts^[7,40]^. The Metware database (MWDB) was employed for the annotation of all metabolites, while quantification was accomplished using the multiple reaction monitoring (MRM) technique. The obtained metabolite data were analyzed using Analyst software (1.6.3)^[41]^. To conduct GC/MS analysis, 500 mg portion of powder was transferred into a 20 mL headspace vial that contained a NaCl-saturated solution to prevent enzymatic reactions. For the analysis using solid-phase microextraction (SPME), each vial was heated to a temperature of 60 °C for a duration of 5 min. Afterward, the sample's headspace was subjected to a 120 µm DVB/CWR/PDMS fiber at 100 °C for a duration of 15 min^[42]^. To conduct the identification and quantification of volatile compounds, an Agilent Model 8890 system coupled with a 7000D mass spectrometer (Agilent Technologies, California, USA) was employed. The MassHunter software was utilized to process the raw data^[43]^.

To explore the relationship between the metabolic profile at different flowering stages, principal component analysis (PCA) was conducted using R software (www.r-project.org). Multiple supervision methods were employed to select an orthogonal partial least-squares discriminant analysis (OPLS-DA) model^[44]^. The OPLS-DA model employed variable importance in projection (VIP) values to evaluate the relative significance of each metabolite across the samples. Metabolites meeting the criteria of VIP ≥ 1 and fold change ≥ 2 or ≤ 0.5 were classified as differential metabolites. Metabolic pathway assignments were carried out utilizing KOBAS2.0, leveraging the KEGG orthology database accessible at http://www.genome.ad.jp/kegg/^[45]^. Moreover, the metabolite content data obtained were subjected to unit variance scaling for normalization purposes. Subsequently, a heatmap was generated using the TBtools software^[46]^.

### RNA sequencing and sequence assembly

The total RNA was extracted following the manufacturer's instructions, utilizing the plant RNA isolation kit (Tiangen, Beijing, China). Quantification of total RNA was conducted, and its quality was evaluated by the Agilent Bioanalyzer 2100. Nine libraries were prepared by utilizing 1.5 µg of total RNA of each sample as the material for subsequent steps. Sequencing of all libraries was conducted on the Illumina HiSeq™ 2,500 platform, resulting in the generation of 150 bp paired-end reads as raw data^[39]^.

In order to acquire processed reads of high quality, the Fastp software (version 0.12) was employed to filter the raw data from all samples^[47]^. The clean reads were subjected to assembly, where they were clustered into expressed sequence tags and de novo assembled into transcripts. The assembly process was conducted by employing Trinity software (version 2.6.6)^[48]^. The reference sequences for subsequent analysis were selected based on the longest sequence within each cluster. Subsequently, the reference sequences were aligned with sequences from publicly available databases, such as Gene Ontology (GO), eukaryotic Orthologous Groups (KOG), Protein family (Pfam), Kyoto Encyclopedia of Genes and Genomes (KEGG), Swiss-Prot protein database (Swiss-Prot), Non-redundant protein sequence database (Nr), and Translated EMBL Nucleotide Sequence database (TrEMBL) using BLAST^[49]^. This allowed us to acquire the relevant functional annotation information.

### Identification of genes exhibiting differential expression (DEGs)

The alignment of clean reads to the reference sequences was performed by RSEM software (version 1.2.26)^[50]^. The expression level of each gene was represented by the FPKM (fragments per kilobase of transcript per million mapped reads) value^[51]^. The DESeq2 R package (version 1.38.2) was employed to detect DEGs across various comparison groups^[52]^. To select significantly DEGs, a threshold of adjusted *P*-value < 0.05 and |log2Fold Change (FC)| ≥ 1 was applied. Functional annotations of the DEGs were performed using GO and KEGG databases. The WEGO 2.0 tool (https://wego.genomics.cn/) and ArigGO were utilized for GO classification, and enrichment analysis^[53]^. To perform KEGG enrichment analyses of the DEGs, the KOBAS 2.0 software was utilized. Moreover, DEGs encoding for transcription factors (TFs) were predicted by hmmscan alignment using the iTAK software^[54]^. The Cytoscape software was utilized to visualize the network of connections between TFs and DEGs^[55]^.

### RT-qPCR analysis

To ensure the reliability of the RNA-seq data, the expression profiles of DEGs were validated using real time quantitative polymerase chain reaction (RT-qPCR) analysis. RNA extraction from various samples was carried out using Tiangen Plant RNA Extraction Kit (Tiangen, Beijing, China). The initial cDNA synthesis was conducted utilizing the PrimeScript RT reagent Kit with gDNA Eraser (Takara, Dalian, China). For RT-qPCR analysis, the ABI 7500 RT PCR system was utilized. The specific primers for the genes were designed using TBtools software^[46]^ (Supplemental Table S1). The following conditions were used for RT-qPCR analysis: an initial denaturation at 94 °C for 30 s, followed by 45 cycles of denaturation at 94 °C for 5 s, annealing at 60 °C for 35 s, and extension at 95 °C for 15 s. A final extension step was performed at 60 °C for 1 min, followed by a melt curve analysis at 95 °C for 15 s. The 2^−ΔΔCᴛ^ method was employed to calculate the relative expression levels of the selected genes^[56]^.

## Results

### Morphological characteristics and hormone concentration changes during flowers development

**Figure 1:**
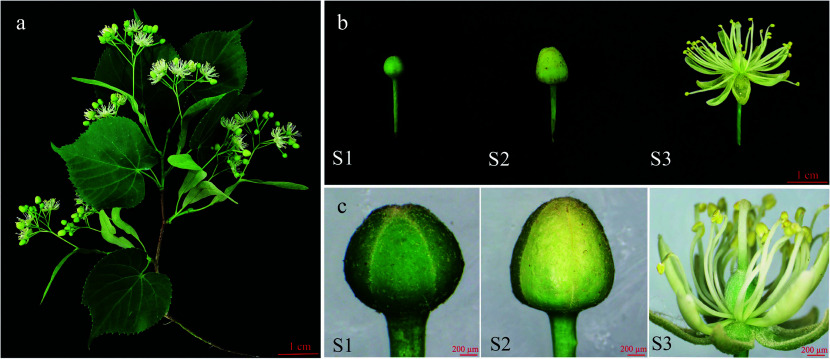
Photographs of *T. amurensis* flowers at different developmental stages. (a) Image of a *T. amurensis* branch with flowers. (b) S1: early bud stage. S2: middle bud stage. S3: fully bloomed stage. (c) S1: microstructure at the early bud stage. S2: microstructure at the middle bud stage. S3: microstructure at the fully bloomed stage. The scale is 200 µm.

In this study, three developmental stages of the flowers (S1, S2, and S3) were assessed. The phenotypes of the different flower development stages are shown in Fig. 1. To observe the microstructure of the flowers, a representative sample was selected from each stage for analysis. The observation results indicated that the S1 stage corresponded to the early bud stage, with small and completely closed petals which were still green. In the S2 flower development stage, the color and size of petals exhibited obvious changes compared to S1. S2 corresponded to the middle bud stage, with large and slightly closed petals that were light yellow. Subsequently, for S3, it corresponded to the full bloom stage, the petals were completely open, and the anthers and stigmata were formed. The findings indicated significant alterations in the flower morphological characteristics during the transition to flowering.

**Figure 2:**
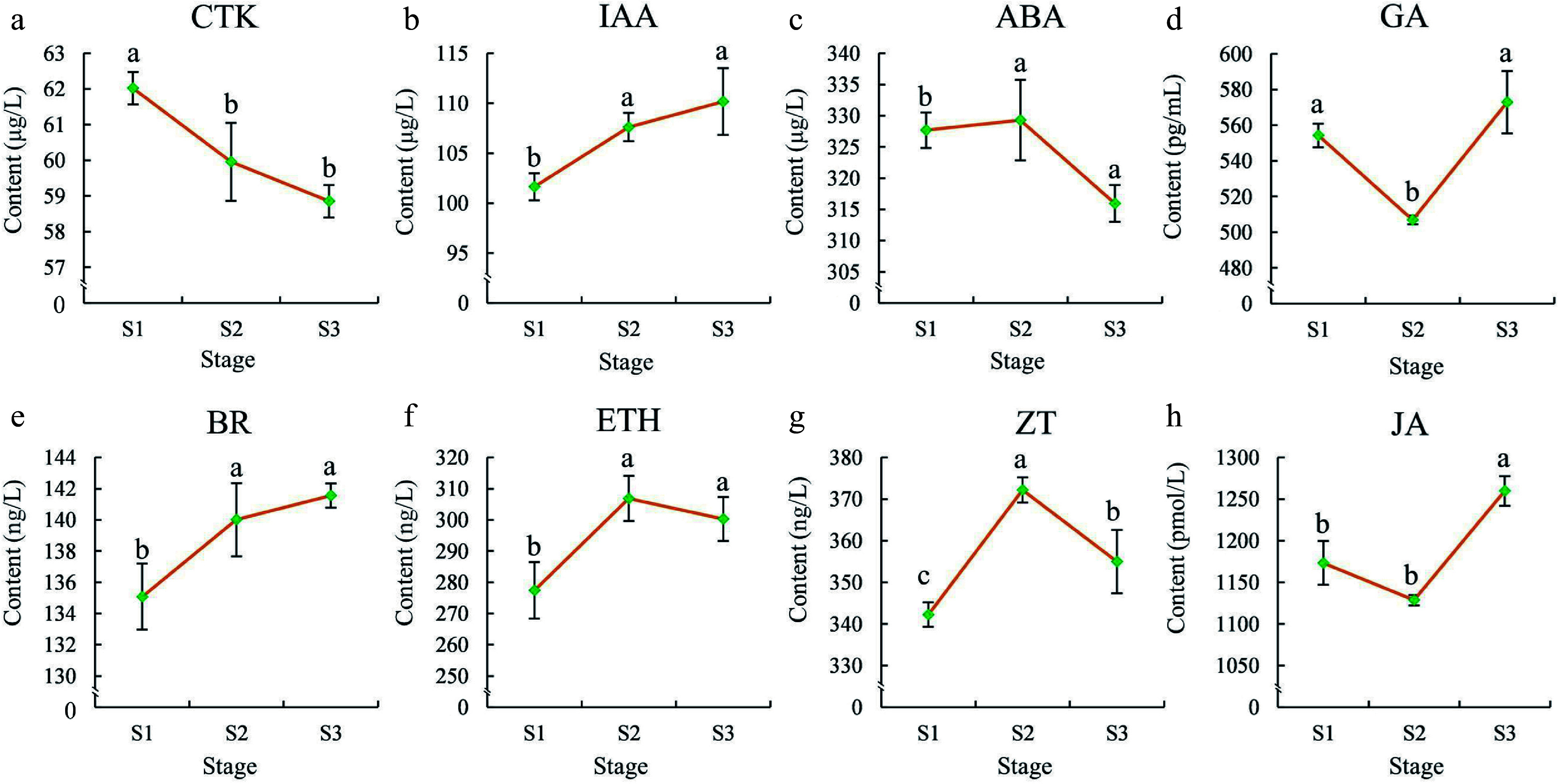
Phytohormone contents in flowers at different stages. (a) CTK, (b) IAA, (c) ABA,(d) GA, (e) BR, (f) ETH, (g) ZT, (h) JA. The error bars in the plot represent the standard error, and the presence of distinct letters on the error bars indicates statistically significant differences at a significance level of *p* < 0.05.

The concentrations of eight endogenous hormones in the flowers were further determined to investigate their roles across the flowering stages. As shown in Fig. 2, the CTK concentration displayed significant differences (*p* < 0.05) between S1 and S2, decreasing progressively from S1 to S3. The GA and JA concentration decreased significantly from S1 to S2 but increased rapidly from S2 to S3 and reached their highest levels in S3. The ABA, ETH, and ZT concentration increased gradually from S1 to S2, showed relatively high levels in S2, and decreased sharply from S2 to S3. Finally, the concentration of BR and IAA increased gradually from S1 to S3, with significant differences observed between S1, S2, and S3 (Fig. 2).

### Sequence assembly and functional annotation

To investigate the alterations in gene expression patterns throughout the three stages of flower development, RNA-seq analysis was implemented. From the RNA-seq analysis, an initial set of 508,883,428 raw reads was obtained. After filtering, 490,842,144 clean reads were retained for further analysis. The clean reads had a cumulative length ranging from 6.59 Gb to 9.24 Gb, averaging at 8.18 Gb in total. The average GC content was determined to be 44.09%, with average percentages of Q20 and Q30 at 96.31% and 91.06% (Supplemental Table S2). After assembly, a total of 407,456 unigenes were obtained, comprising 298,619,887 bp. The N50 value was 904 bp, with an average unigene length of 733 bp (Supplemental Table S3). Furthermore, 229,545 (56.3%) unigenes had a length exceeding 500 bp, and a total of 15,032 (20.5%) unigenes surpassed 1,000 bp in length (Supplemental Table S4). On average, the alignment rate of clean reads to the reference sequence was approximately 82.34% (Supplemental Table S5).

**Figure 3:**
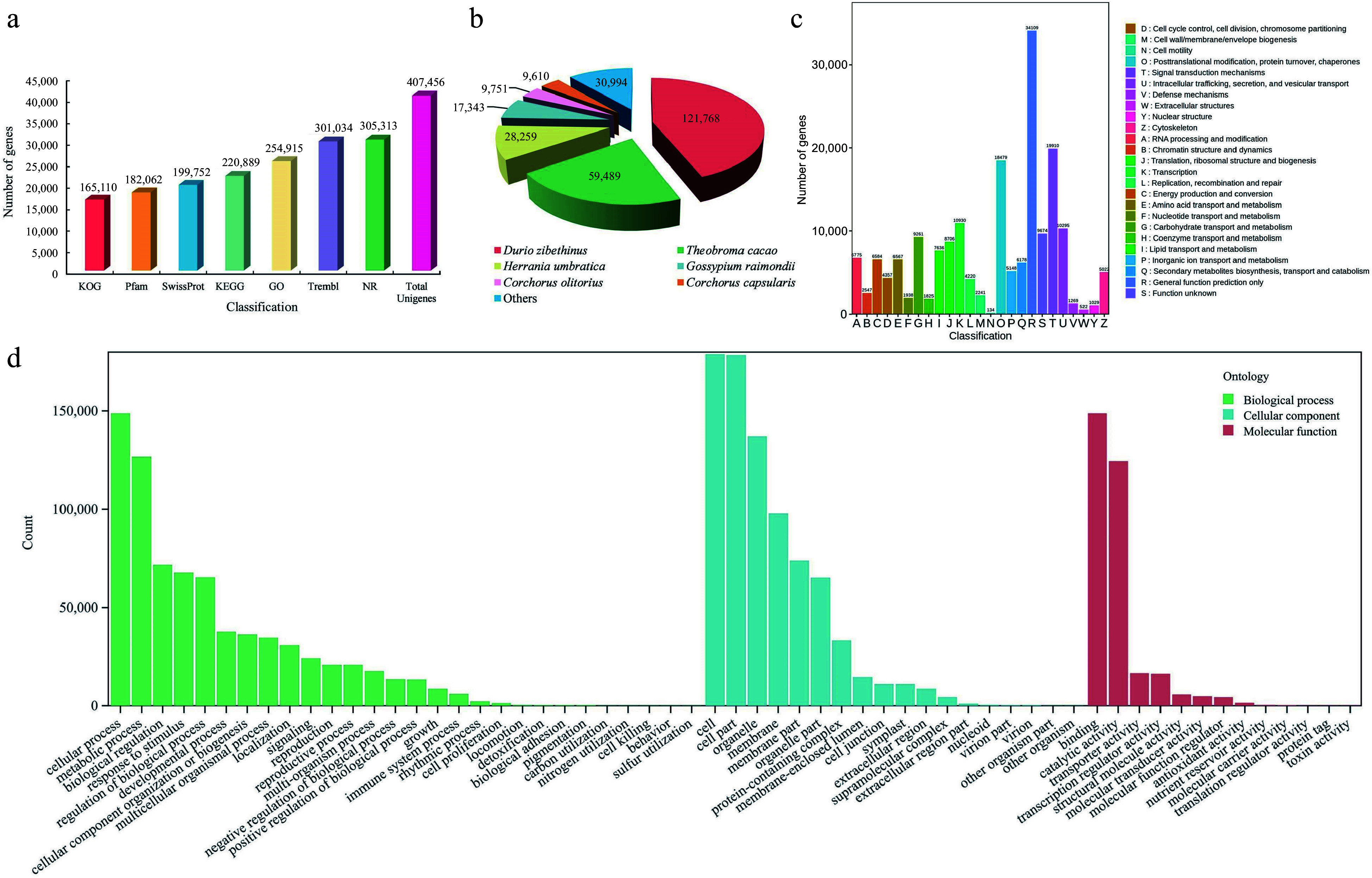
Identification and annotation analysis of unigenes of *T. amurensis* flowers. (a) Annotation information of unigenes in various public databases. (b) Statistical analysis of species distribution from the Nr database. (c) Function annotation of unigenes in KOG database. (d) The distribution across functional categories of unigenes in GO database.

To further investigate the functional annotations of the unigenes after assembly, alignments were performed with sequences from seven public functional databases. Out of the total 307,094 unigenes, at least one function was annotated in the public databases. Among the databases used for annotation, the Nr database contained the largest count of annotated unigenes (305,313), followed by TrEMBL (301,034). In contrast, the KOG database had the lowest number of annotated unigenes (165,110) (Fig. 3a and Supplemental Table S6). The unigenes were strongly matched to gene sequences from *Durio zibethinus* (121,768), *Theobroma cacao* (59,489), and *Herrania umbratica* (28,259) (Fig. 3b). Moreover, 165,110 unigenes were annotated to 25 categories of the KOG protein database, mainly to the (R) ''General function prediction only'' (34,109), (T) ''Signal transduction mechanisms'' (19,910), and (O) '' Posttranslational modification, protein turnover, chaperones'' (18,479) categories (Fig. 3c). The unigenes were annotated to three categories, namely biological process (BP), molecular function (MF), and cellular component (CC), for the purpose of conducting GO analysis. Regarding the BP categories, unigenes were mainly associated with the cellular process (148,751), metabolic process (126,677), and biological regulation (71,579) categories. Regarding the MF categories, binding (148,687), catalytic activity (124,492), and transporter activity (16,531) were the most represented functions. Finally, in the CC categories, unigenes were mainly associated with the cell (178,918), cell part (178,486), and organelle (137,042) ontologies (Fig. 3d).

### Identification and enrichment analysis of DEGs

**Figure 4:**
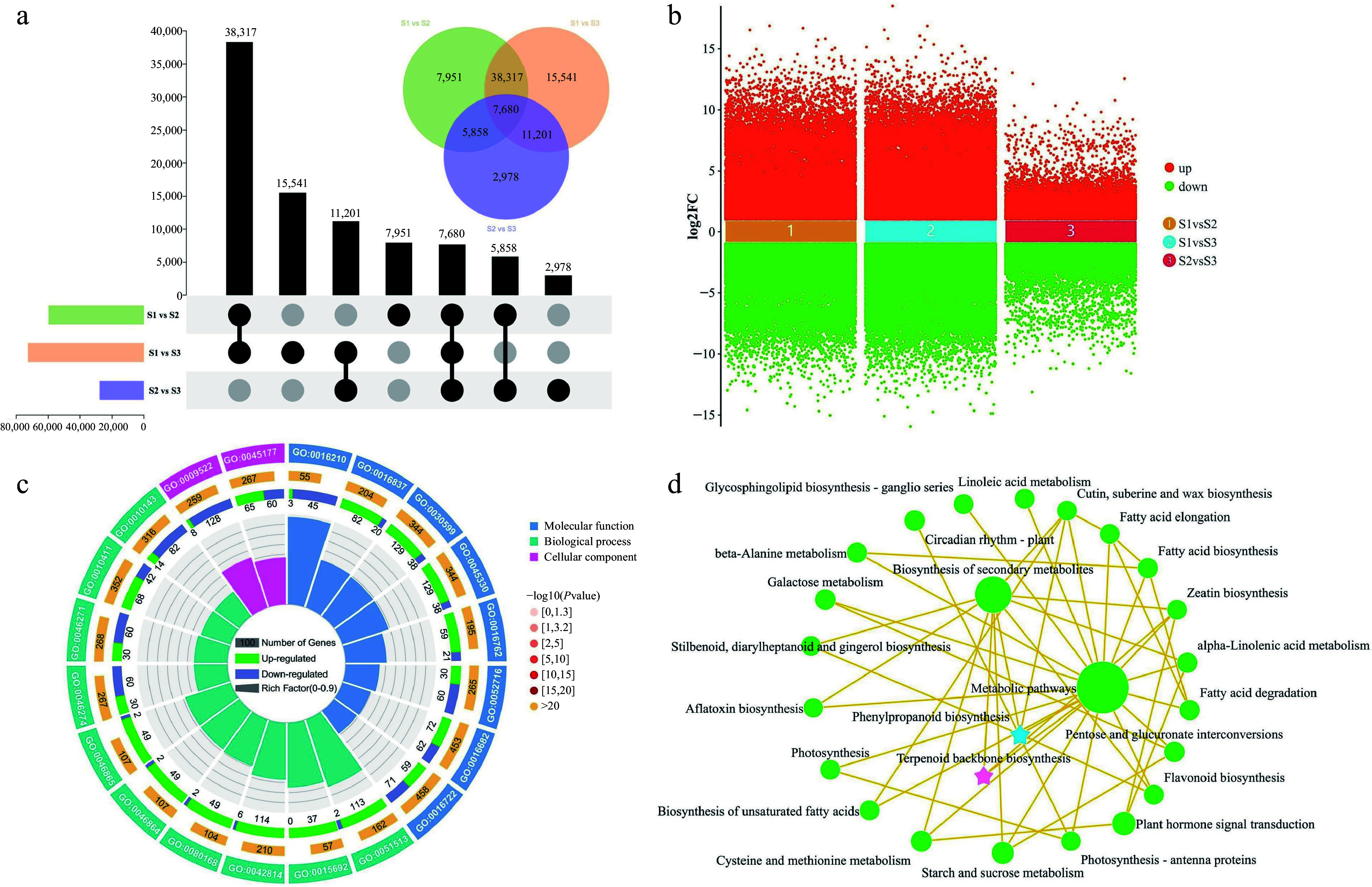
Identification and enrichment analysis of DEGs in three comparison groups. (a) UpSet and Venn diagram of DEGs in three comparison groups. (b) Volcano plot of upregulated and downregulated DEGs in three comparison groups. (c) Gene Ontology enrichment circle diagram of DEGs. (d) KEGG enrichment analysis network diagram of DEGs.

In the pursuit of identifying DEGs during the course of flower development, a comparison was made between the transcript levels of each unigene across various stages of flower development. The comparison between stages S1 and S3 revealed the highest number of DEGs (72,739), including 35,427 that were upregulated and 37,312 that were downregulated. In the S1 vs S2 comparison, 59,806 DEGs (29,228 upregulated and 30,578 downregulated) were identified, and in the S2 vs S3 comparison, 27,717 DEGs were identified (14,577 upregulated and 13,140 downregulated) (Fig. 4a & b). Among the different developmental stages, there were 7,680 DEGs that were commonly expressed. To illustrate the overlap of these DEGs among the three groups, a Venn diagram was employed for visualization (Fig. 4a). The results revealed the genes that exhibited changes in expression levels during flower development.

To elucidate the biological functions of DEGs identified in the comparative transcriptome analysis, functional annotations were assigned to the genes through the use of GO and KEGG analyses. The analysis of the top 20 GO terms revealed significant enrichment of DEGs across the categories of BP, CC, and MF. The main enriched functions consisted of ''regulation of monopolar cell growth'' (GO:0051513), ''oxidoreductase activity, oxidizing metal ions'' (GO:0016722), ''phenylpropanoid catabolic process'' (GO:0046271), and ''terpenoid transport'' (GO:0046865) (Fig. 4c). Subsequently, KEGG enrichment analysis was carried out to investigate the most highly represented metabolic and signal transduction pathways among the DEGs during the flower development of *T. amurensis*. The DEGs demonstrated notable enrichment in several KEGG pathways, encompassing ''metabolic pathways'', ''biosynthesis of secondary metabolites'', ''plant hormone signal transduction'', ''phenylpropanoid biosynthesis'', and ''terpenoid backbone biosynthesis'' (Fig. 4d). Significantly, the GO and KEGG enrichment analyses consistently unveiled significant enrichment of pathways related to terpenoid and phenylpropanoid biosynthesis. These findings provide compelling evidence for the significant involvement of genes associated with terpenoid and phenylpropanoid biosynthesis in the process of flower development.

### Differentially expressed metabolites (DEMs) analysis and metabolic pathway enrichment

**Figure 5:**
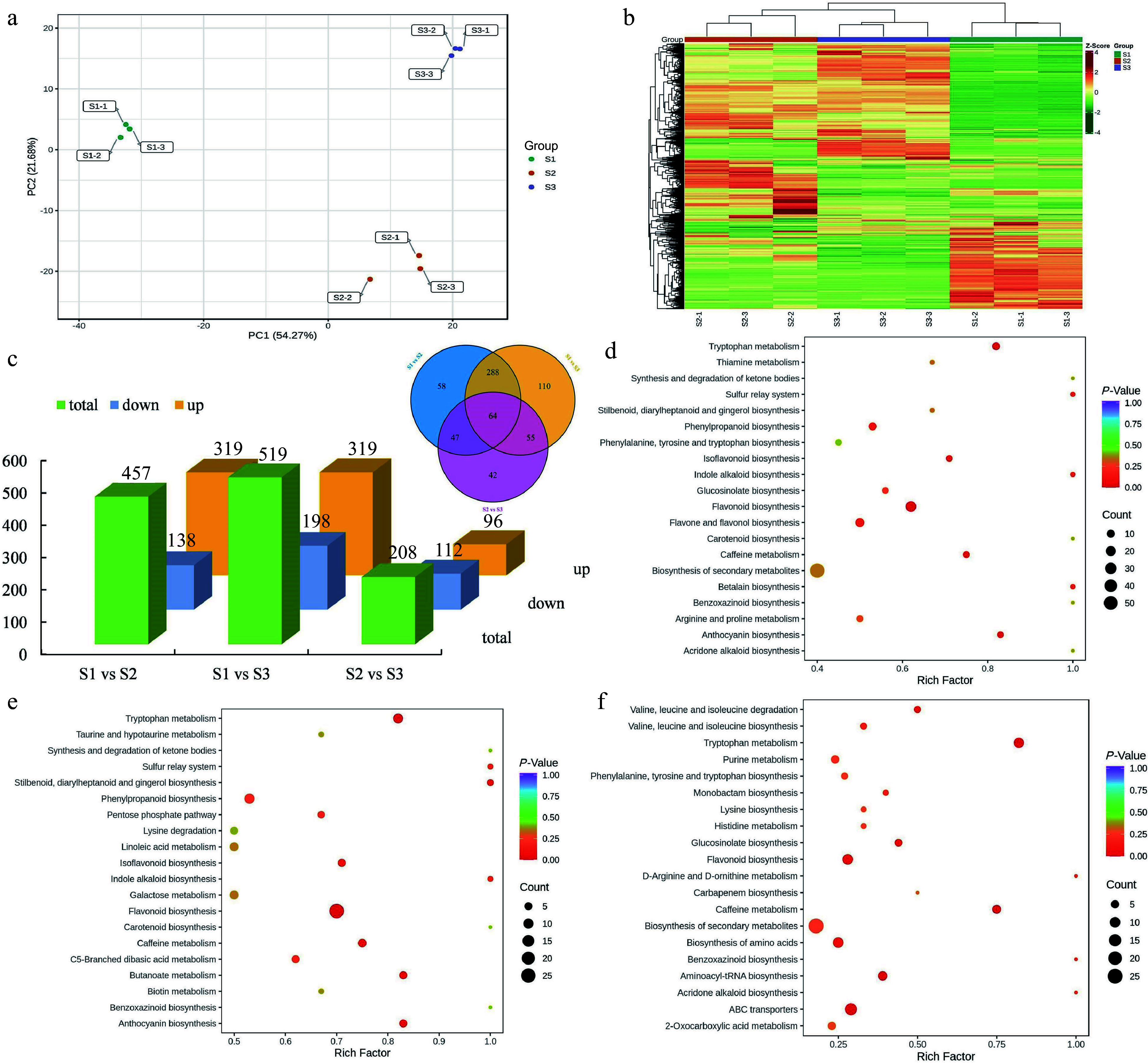
Identification and enrichment analysis of DEMs. (a) PCA of the metabolome data from three stages. (b) Cluster heatmap of all DEMs in three stages. (c) Statistical analysis of upregulated and downregulated DEMs in three comparison groups. (d) KEGG enrichment analysis of the DEMs in the S1 vs S2 group. (e) KEGG enrichment analysis of the DEMs in the S1 vs S3 group. (f) KEGG enrichment analysis of the DEMs in the S2 vs S3 group.

To comprehensively analyze the differential expression and functional significance of metabolites across different stages of flowering, a comprehensive detection of metabolites was performed. A total of 664 metabolites were detected and identified throughout the various stages of flower development. These DEMs mainly comprising flavonoids (189), phenolic acids (105), lipids (74), amino acids and derivatives (59), organic acids (43), nucleotides and derivatives (39), alkaloids (36), lignans and coumarins (35), terpenoids (26), tannins (10) and other metabolites (48) (Supplemental Table S7). Principal component analysis (PCA) demonstrated distinct variations in the overall metabolite profiles among the S1, S2, and S3 groups, whereas the within-group sample variability was minimal (Fig. 5a). Furthermore, hierarchical clustering analysis was implemented to examine the patterns of metabolite accumulation across samples. The findings revealed consistent division of all samples into three distinct groups, aligning with the results obtained from PCA. Notably, specific metabolites exhibited higher concentrations during the S2 and S3 stages, suggesting their significant involvement in flower development (Fig. 5b). The comparative analysis between S1 and S2 revealed a total of 457 DEMs, with 319 upregulated and 138 downregulated. Similarly, in the S1 vs S3 and S2 vs S3 comparison groups, 517 and 208 DEMs were found, respectively. In addition, a shared group of 64 metabolites exhibited differential expression across all three comparison groups, indicating their consistent modulation throughout the various developmental stages (Fig. 5c).

Moreover, KEGG enrichment analysis was performed for DEMs. In the comparison between S1 and S2 groups, the DEMs were categorized into 74 KEGG-enriched pathways. Similarly, in the S1 vs S3 and S2 vs S3 comparisons, the overrepresented metabolic pathways consisted of 80 and 49 pathways, respectively. Notably, the KEGG pathways of ''biosynthesis of secondary metabolites'', ''phenylpropanoid biosynthesis'', and ''flavonoid biosynthesis'' were consistently identified as the shared and highly enriched pathways in the group comparisons of S1 vs S2 (Fig. 5d), S1 vs S3 (Fig. 5e), and S2 vs S3 (Fig. 5f). In addition, ''ubiquinone and other terpenoid-quinone biosynthesis'', ''starch and sucrose metabolism'', and ''phenylalanine metabolism'' were also overrepresented in the three comparison groups. According to the statistical analysis of enriched DEGs and DEMs, we observed significant enrichment of pathways related to terpenoid and phenylpropanoid biosynthesis. Overall, these results further confirmed that the DEMs involved in terpenoid and phenylpropanoid biosynthesis pathways were significantly altered during flower development in *T. amurensis*.

### DEGs are involved in hormone biosynthesis and signal transduction in the different flower development stages

**Figure 6:**
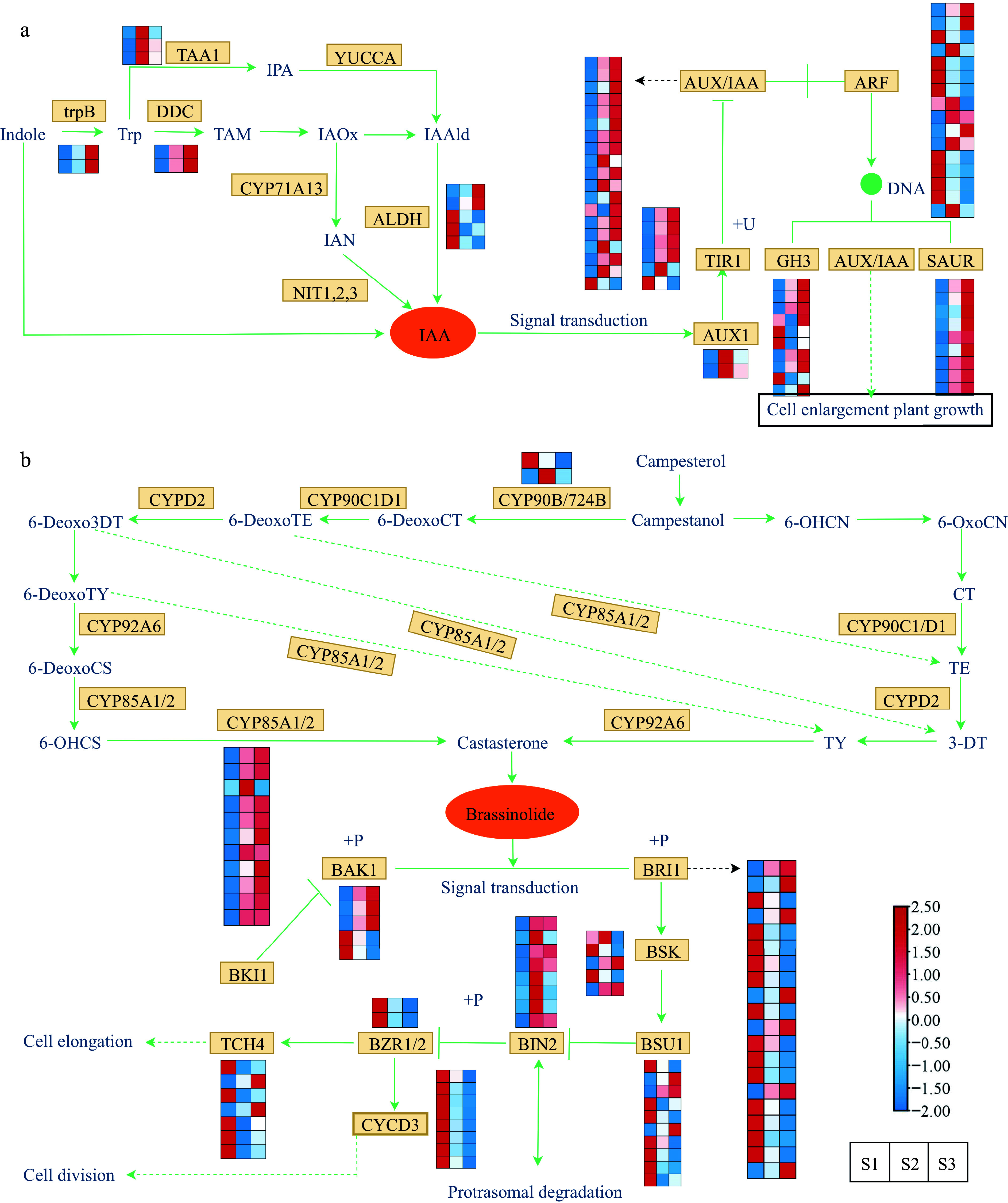
Expression profiles of DEGs associated with phytohormone biosynthesis and signal transduction. (a) Expression profiles of DEGs involved in the auxin. (b) Expression profiles of DEGs involved in the brassinosteroid. The expression profiles of genes at the three stages are displayed in separate columns within the colored boxes, while each row represents a distinct gene. The color gradient indicates the expression levels, with red indicating upregulation and blue indicating downregulation. trpB, tryptophan synthase beta chain; DDC, tryptophan decarboxylase; TAA, tryptophan aminotransferase; YUCCA, indole-3-pyruvate monooxygenase; CYP71A13, indoleacetaldoxime dehydratase; ALDH, aldehyde dehydrogenase; NIT, nitrilase; AUX1, auxin influx carrier 1; TIR1, transport inhibitor response 1; AUX/IAA, auxin-responsive protein IAA; ARF, auxin response factor; GH3, gretchen hagen 3; SAUR, small auxin upregulated RNA; CYP90B/724B, steroid 22-alpha-hydroxylase; CYP92A6, typhasterol/6-deoxotyphasterol 2alphahydroxylase; CYP85A1/2, brassinosteroid-6-oxidase 1/2; BRI1; BRI1 kinase inhibitor 1; BAK1, brassinosteroid insensitive 1-associated receptor kinase 1; BSK, BR-signaling kinase; BSU1, serine/threonine-protein phosphatase; BIN2, brassinosteroid insensitive 2; BZR1/2, brassinosteroid resistant 1/2; TCH4, xyloglucan: xyloglucosyl transferase; CYCD3, cyclin D3.

Given the observed elevation in concentrations of IAA and BR throughout flower development, we proceeded with an in-depth examination of potential structural genes linked to the synthesis and signal transduction pathways of IAA and BR. Heat maps were generated to investigate the gene expression, incorporating the annotation information of the DEGs obtained from diverse public databases. IAA is the most abundant auxin in plants. By regulating the expression of structural genes in the auxin signaling pathway, it also indirectly affects plant growth and development^[57]^. Under the influence of different enzymes, IAA is synthesized through both the tryptamine (TAM) pathway and the indole pyruvic acid (IPA) pathway, starting from tryptophan (Trp). Moreover, IAA can also be synthesized *via* the indole-3-acetaldoxime (IAOx) pathway through the key intermediate indole acetonitrile (IAN) *via* the catalytic activity of indoleacetaldoxime dehydratase (CYP71A13) and nitrilase (NIT). In this study, 12 DEGs were identified, including the genes of *TAA1*, *trpB*, *DDC*, and *ALDH* family (Fig. 6a). The expression of *trpB* and *DDC* genes was upregulated in S2 and S3, suggesting their key role in auxin synthesis. The *ALDH* gene family had both up/downregulated genes at different flowering stages. Through differential gene expression screening, 67 DEGs, including the genes of *ARF*, *AUX/IAA*, *GH3*, *TIR1*, *SAUR*, and *AUX1* family, were detected in this pathway (Fig. 6a & Supplemental Table S8). In the examination of gene expression patterns, it was discovered that the *TIR1*, *GH3*, *ARF*, and *AUX/IAA* gene families exhibited a combination of up-regulated and down-regulated genes throughout all three flowering stages.

Brassinolide, a steroid hormone, acts as a plant growth regulator and is crucial for embryo, flower, and fruit development^[58]^. Regarding the BR synthesis pathway (Fig. 6b & Supplemental Table S9), we found two families (*CYP90B/724B* and *CYP85A1/2*) of genes that were differentially expressed at different stages of flower development. Significantly, within the *CYP85A1/2* family, all genes exhibited elevated expression levels during S2 and S3 stages, with the exception of the gene *Cluster-20864.178597*. Moreover, the transmission of BR signals from the receptor BRI1 to the transcription factor *BZR1* involves a cascade of phosphatases and kinases, including BAK1, BRI1, BSK, BIN2, TCH4, CYCD3, BSU1, and BZR1/2^[59]^. The genes encoding *BAK1*, *BRI1*, *BSK*, *TCH4*, and *BSU1* showed both up/downregulation during flower development, while the expression levels of *CYCD3* and *BZR1/2* family genes were downregulated of S2 and S3 stages. Specifically, the expression profiles of genes belonging to the *BIN2* family were significantly elevated in the S2 stage, suggesting their potential role in promoting cell division during flower development.

### Analyses of the DEGs and DEMs involved in the terpenoid biosynthesis pathway during flowering

**Figure 7:**
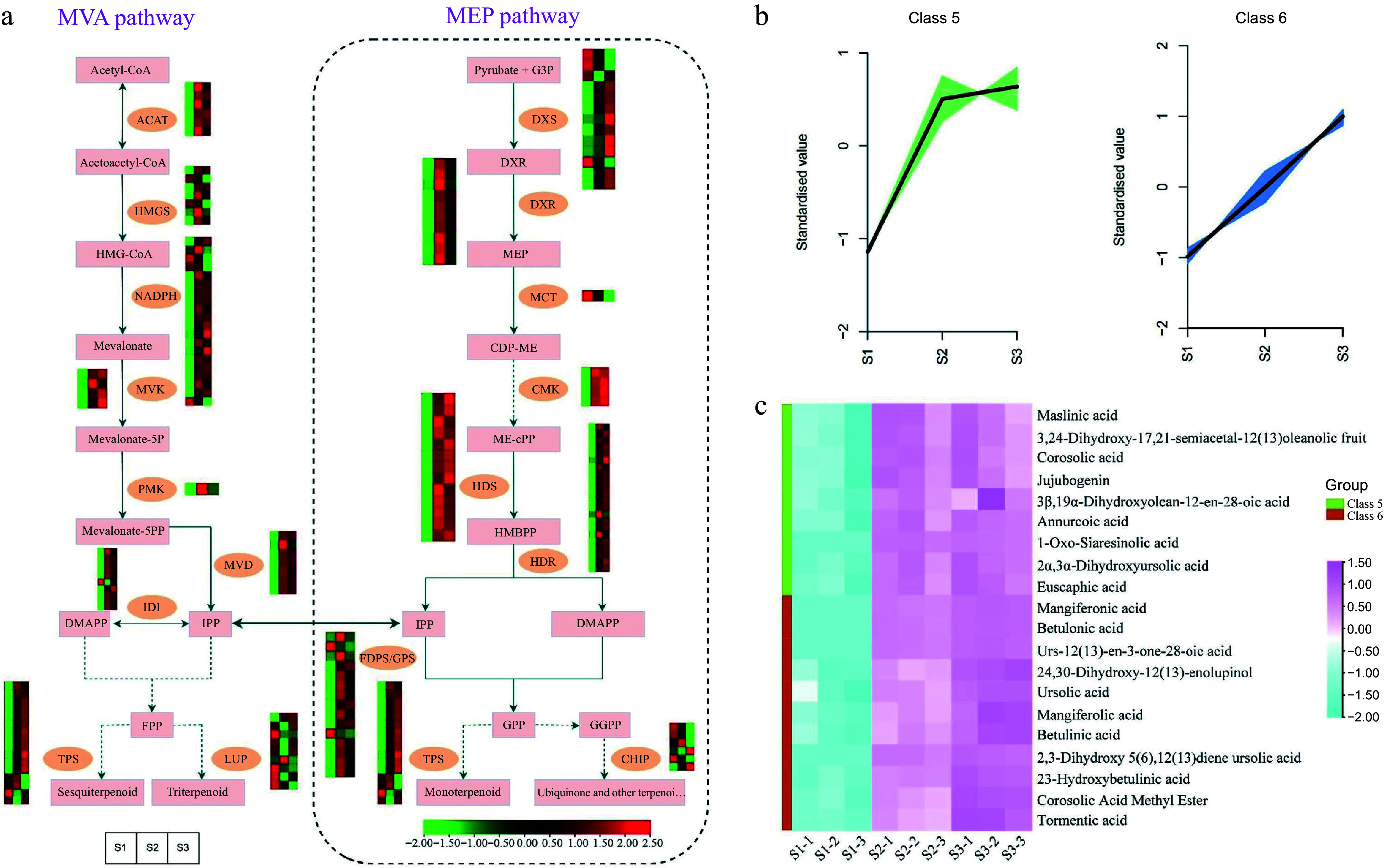
Illustrations and expression patterns of DEGs and DEMs implicated in the biosynthesis pathway of terpenoids. (a) Expression levels of DEGs. (b) K-means cluster analysis of DEM accumulation. (c) Heatmap of DEMs in the terpenoid biosynthesis pathway. The expression profiles of genes at the three stages are displayed in separate columns within the colored boxes, while each row represents a distinct gene. The color gradient indicates the expression levels, with red indicating upregulation and green indicating downregulation. ACAT, acetyl-CoA C-acetyltransferase; HMGS, 3-hydroxy-3-methyl-glutaryl-CoA synthase; MVK, mevalonate kinase; PMK, phosphomevalonate kinase; MVD, mevalonate-5-pyrophosphate decarboxylase; IDI, isopentenyl-diphosphate delta-isomerase; LUP, lupeol synthase; DXS, 1-deoxy-D-xylulose 5-phosphate synthase; DXR, 1-deoxy-D-xylulose-5-phosphate reductoisomerase; MCT, 2-C-methyl-D-erythritol 4-phosphate cytidylyltransferase; CMK, 4-diphosphocytidyl-2-C-methyl-D-erythritol kinase; HDS, 1-hydroxy-2-methyl-2-butenyl 4-diphosphate synthase; HDR, 1-hydroxy-2-methyl-2-butenyl 4-diphosphate reductase; FDPS/GPS, farnesyl diphosphate synthase/geranyl diphosphate synthase; TPS, terpene synthases.

The biosynthesis pathway responsible for producing ''terpenoid backbones'' involves both the mevalonate (MVA) pathway and the 2-C-methyl-D-erythritol 4-phosphate (MEP) pathway. These pathways are responsible for the synthesis of monoterpenes and sesquiterpenes, respectively^[60]^. To enhance our comprehension of the molecular mechanisms behind the variations in aroma during *T. amurensis* flower development, the expression profiles of 165 DEGs associated with the MVA and MEP pathways, which are key pathways for terpene synthesis, were analyzed (Fig. 7a & Supplemental Table S10). Among them, only one DEG corresponded to *PMK* and *MCT*, while more than one DEG corresponded to *ACAT*, *HMGS*, *DXR*, and *TPS*. Moreover, 165 terpene biosynthesis pathway genes were significantly differentially expressed. Certain DEGs, such as genes encoding *LUP*, *DXS*, and *CHIP*, had low expression in S2 and S3 stages and high expression in the S1 stage, whereas others showed the opposite pattern (e.g., genes encoding *ACAT*, *NADPH*, and *HDR*). During flower development, particularly in the middle and late stages (S2 and S3), the expression profiles of the majority of DEGs related to terpene synthesis exhibited a substantial increase. This observation suggests that these stages are crucial for terpenoid production in flowers.

To gain a deeper understanding of the dynamics of DEMs accumulation, a K-means cluster analysis was conducted utilizing the relative contents of metabolites throughout the S1, S2, and S3 flowering stages. Throughout flowering, the accumulation patterns of the DEMs displayed various trends. Remarkably, during the S2 and S3 stages, DEMs assigned to cluster 5 and cluster 6, respectively, demonstrated considerably higher accumulation levels compared to the S1 stage (Fig. 7b). Intriguingly, the observed accumulation patterns of these DEMs closely mirrored the expression changes observed in the majority of genes associated with the terpenoid synthesis pathway during flower development. Terpenoids are key metabolites and important constituents of floral odors in many plants. Therefore, the DEMs in Cluster 5 and Cluster 6 were further investigated to identify the major terpenoids produced during the different flowering stages. 20 DEMs belonging to triterpenoids were found in the two clusters, including maslinic acid, corosolic acid, and jujubogenin, etc. They showed high accumulation levels in the S2 and S3 flowering stages (Fig. 7c & Supplemental Table S11). This suggests that the accumulation of these metabolites may directly affect the aroma production and profile in the flowers of *T. amurensis*.

### Identification and expression analysis of DEGs and DEMs in the phenylpropanoid biosynthesis pathway during flowering

**Figure 8:**
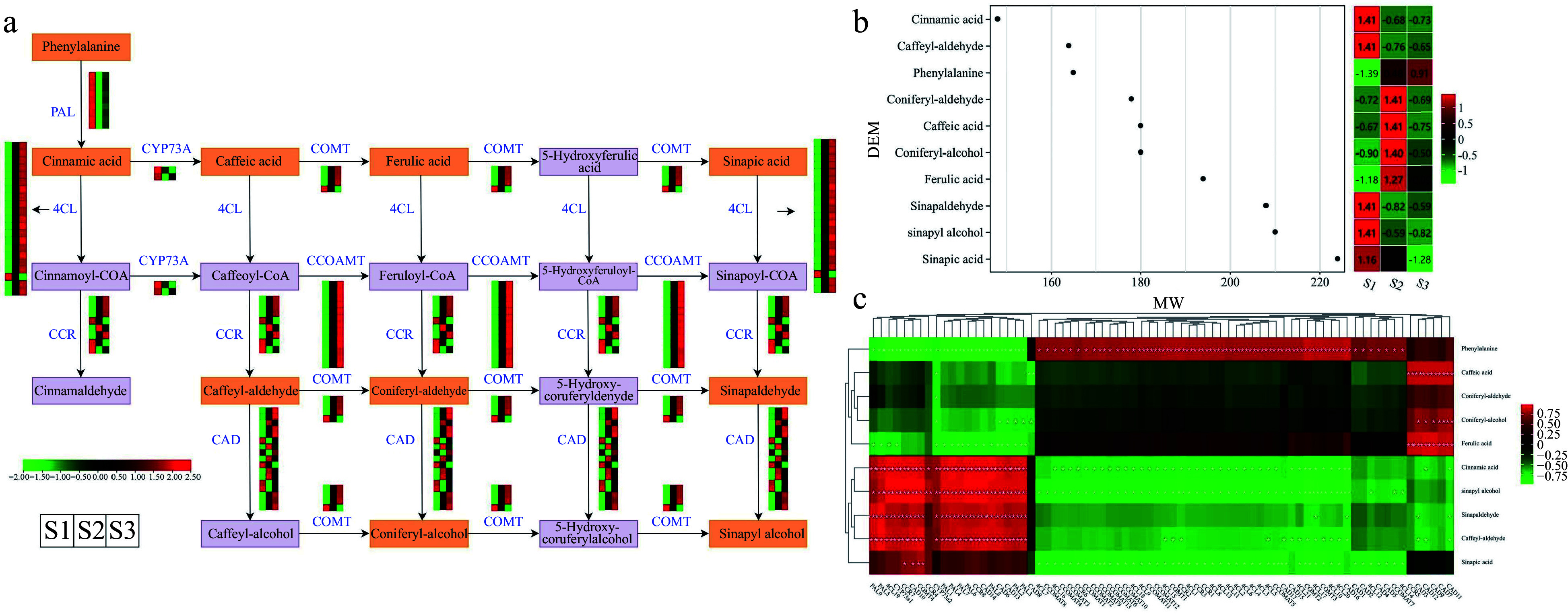
Illustrations and expression patterns of DEGs and DEMs involved in the phenylpropanoid biosynthesis pathway. (a) Expression levels of DEGs. (b) Heatmap of DEMs in the phenylpropanoid biosynthesis pathway. (c) Correlation analysis of phenylpropanoid biosynthesis-related structural gene expression profile and DEM accumulation. The expression profiles of genes at the three stages are displayed in separate columns within the colored boxes, while each row represents a distinct gene. The color gradient indicates the expression levels, with red indicating upregulation and green indicating downregulation. PAL, phenylalanine ammonia-lyase; 4CL, 4-coumarate–CoA ligase; CCR, cinnamoyl-CoA reductase; COMT, caffeic acid 3-O-methyltransferase; CCOAMT, caffeoyl-CoA O-methyltransferase; CAD, cinnamyl-alcohol dehydrogenase; CYP73A, trans-cinnamate 4-monooxygenase.

The phenylpropanoid biosynthesis pathway, accountable for generating phenylpropanolamine and various other compounds, serves crucial functions in plant growth, defense mechanisms, and acts as a primary pathway governing aroma production^[30]^. The KEGG enrichment analysis revealed an overrepresentation of the 'phenylpropanoid biosynthesis' pathway in three comparison groups, based on the analysis of DEGs and DEMs. We investigated the expression profiles of crucial genes and the accumulation of metabolites associated with the phenylpropanoid biosynthesis pathway. The analysis revealed that a combined total of ten DEMs (including sinapic acid, phenylalanine, and ferulic acid, etc.) and 74 DEGs (including nine *PALs*, eight *CCRs,* and four *COMTs*, etc.) exhibited distinct expression patterns throughout flower development (Fig. 8a & Supplemental Table S12). Heatmap analysis revealed that certain structural genes, such as one gene (*Cluster-20864.233536*) encoding for *CCR* and five genes (*Cluster-20864.112764*, *Cluster-20864.271610*, *Cluster-20864.253472*, *Cluster-20864.220953*, and *Cluster-20864.76579*) encoding for *CADs*, exhibited particularly elevated expression levels during the S2 stage in flowers. Moreover, apart from *Cluster-20864.148160*, the expression profiles of genes encoding *4CL* and *CCOMT* were elevated in the S2 and S3 developmental stages, in contrast to the S1 stage.

Based on the heatmap illustrating the accumulation levels of ten DEMs (with molecular weights between 145 and 225), the accumulation of ferulic acid, coniferyl-alcohol, caffeic acid, and coniferyl-aldehyde demonstrated a substantial elevation in the S2 stage, when compared to the S1 stage (Fig. 8b & Supplemental Table S13). Notably, phenylalanine accumulation exhibited a gradual upward trend, with the highest accumulation levels reaching during the S3 stage. In addition, we conducted a correlation analysis to explore potential regulatory relationships between the 10 DEMs and structural genes in the phenylpropanoid biosynthesis pathway (Fig. 8c & Supplemental Table S14). The results illustrated a noteworthy positive association between the expression levels of *PALs*, *4CLs*, *CCRs*, and *CADs*, and the accumulation of cinnamic acid, sinapyl alcohol, sinapaldehyde, and caffeyl-aldehyde. Notably, four DEMs (caffeic acid, coniferyl-aldehyde, coniferyl-alcohol, and ferulic acid) showed positive correlations only with CAD-related genes (including *CAD7*, *CAD9*, and *CAD12*). The findings suggested that the DEGs and DEMs identified have significant functions in the phenylpropanoid biosynthesis pathway and are probably key contributors to the flower's aroma accumulation in *T. amurensis*.

### Identification of transcription factors (TFs) and network analysis of structural genes involved in aroma formation

**Figure 9:**
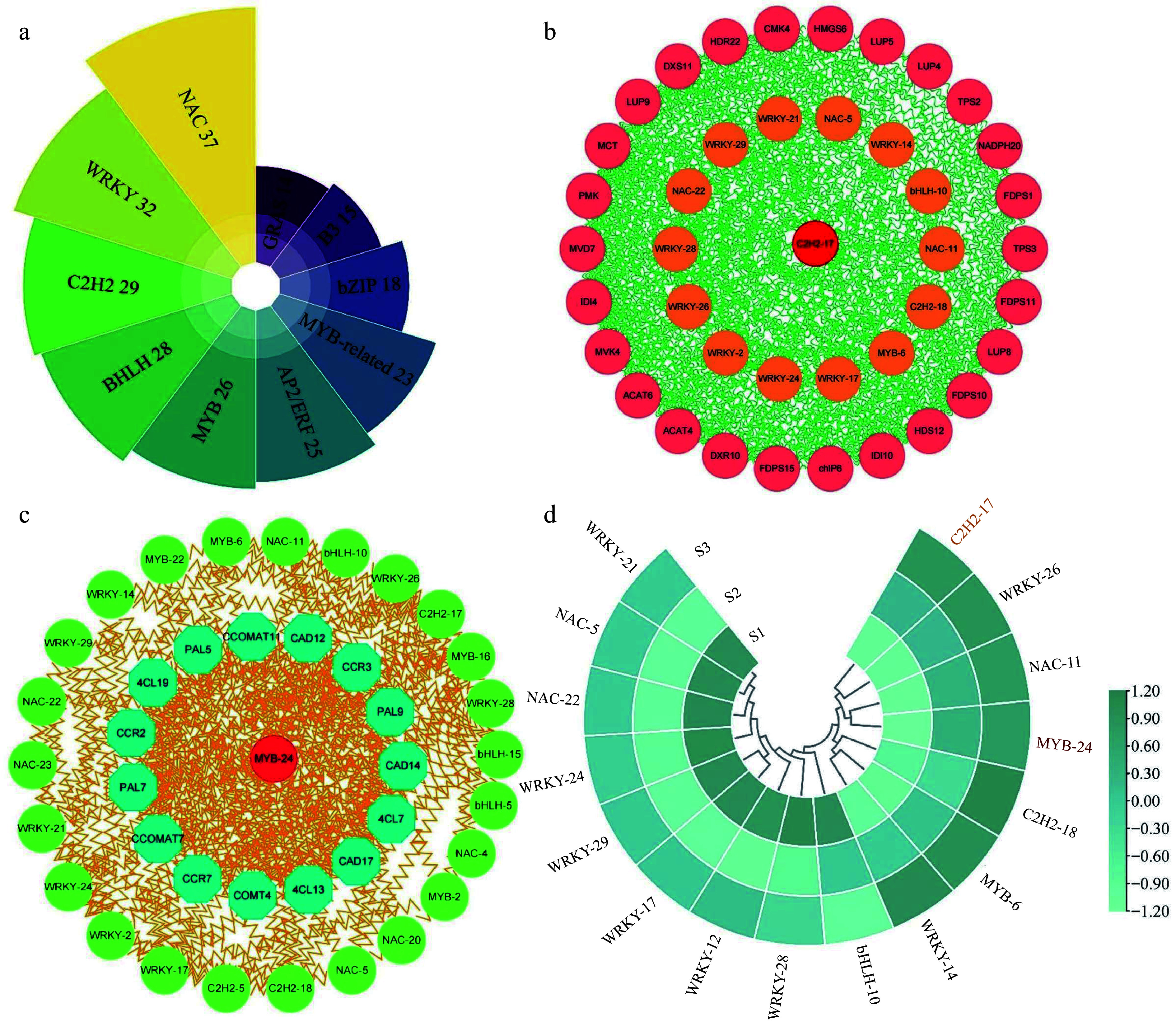
Analysis of correlation and expression patterns between structural genes and transcription factors. (a) Statistical analysis of the highest number of ten transcription factor families in three comparison groups. (b) Correlation network analysis of terpenoid biosynthesis-related structural genes and the highest number of five transcription factor families. (c) Correlation network analysis of phenylpropanoid biosynthesis-related structural genes and the top five transcription factor families. (d) The expression patterns of 16 selected transcription factors.

Apart from key structural genes, TFs play a vital role in directly modulating the expression profiles of multiple genes and metabolic pathways, consequently facilitating the generation and emission of floral fragrance. In current study, we identified 409 TFs from the transcriptome data, distributing across 45 families. Among these, the highest number of TFs were distributed in the *NAC* (37), *WRKY* (32), *C2H2* (29), and *bHLH* (28) families, among others (Fig. 9a & Supplemental Table S15). To explore the regulatory connections among the identified TFs, genes associated with terpenoid metabolism, and genes involved in phenylpropanoid biosynthesis, a co-expression network was established. For this analysis, homologous proteins of *Arabidopsis* were sourced from STRING database.

The network depicted in Fig. 9b integrated genes related to terpenoid metabolism and the top five TF families among the DEGs. Notably, the analysis revealed robust interactions between these TFs and the genes involved in terpenoid metabolism. Notably, *MCT*, *MVD7*, *DXS11*, *HDR22*, and *LUP9* exhibited significant interaction with TFs, implying their potential roles in regulating aroma formation in *T. amurensis* flowers. Moreover, we discovered that the *C2H2-17* exhibited the most numerous expressions with other genes. This may indicate that it functions as the central regulator in the network. A second network (Fig. 9c) revealed the relationship between TFs and genes associated with phenylpropanoid biosynthesis. Among the genes present in the network, *CAD12*, *PAL9*, and *4CL13* exhibited notable interactions with TFs in comparison to the remaining genes. Unlike the terpenoid metabolism regulatory network, our findings indicated that *MYB-24* exhibited the strongest connectivity with phenylpropanoid biosynthesis-related genes, thus considered the hub regulator of phenylpropanoid biosynthesis. Interestingly, multiple shared transcription factors were present in both networks, including *WRKY-21*, *NAC-5*, *NAC-22*, and *WRKY-24*, etc, and strongly correlated with terpenoid metabolism and phenylpropanoid biosynthesis genes.

Sixteen TFs from the co-expression network were chosen for further investigation, and their expression patterns were assessed in the different stages (Fig. 9d). The expression levels of five shared TFs, namely *WRKY-26*, *NAC-11*, *C2H2-18*, *MYB-6*, and *WRKY-14* were notably high during the S2 and S3 stages of flower development. Furthermore, in the S2 and S3 stages, two central regulators, *C2H2-17* and *MYB-24*, displayed pronounced expression, further supporting their essential role in the modulation of floral fragrance development. Certain genes (including *WRKY-21*, *NAC-5*, *NAC-22*, and *WRKY-24*) declined their expression profiles in S2 and S3 stages relative to the S1 stage, suggesting that they may be negative regulators of flower growth and development. These results indicate that transcription factors have strong interactions with genes related to floral scent metabolism and are an indispensable component of floral scent synthesis.

### Validation of RNA-Seq data by RT-qPCR

A total 14 DEGs were chosen for RT-qPCR assays to confirm the reliability of the RNA-seq data. Among them, five DEGs (*Cluster-20864.228631*, *Cluster-20864.206192*, *Cluster-20864.167641*, *C2H2-17*, and *MYB-24*) are transcription factors, three DEGs (*Cluster-20864.198501*, *Cluster-20864.140634*, *Cluster-20864.162042*) are associated with the terpenoid biosynthesis pathway, three DEGs (*Cluster-20864.210350*, *Cluster-20864.215451*, *Cluster-20864.197278*) are related to the phenylpropanoid biosynthesis pathway, while the rest are ordinary differentially expressed genes. The results demonstrated a high degree of agreement between the RNA-seq data and qPCR data in terms of gene expression patterns, exhibiting a correlation coefficient of 0.8584 (Supplemental Figs S1, S2, & S3). These results provide evidence of the dependable nature of the transcriptomic data.

## Discussion

### Alterations in phytohormone levels and their regulatory functions of flower development

Phytohormones assume a central role in governing plant development, orchestrating diverse processes including cell division, cell expansion, differentiation, as well as flower and fruit development^[61]^. Flower development involves various plant hormones, which, synergistically or antagonistically, influence flower size, shape, color, and fragrance^[62,63]^. Previous research has provided evidence supporting the role of auxin in promoting the growth and expansion of floral structures, as well as its ability to stimulate the synthesis of fragrance compounds^[64,65]^. A report indicates that the phytohormones GA and JA act as growth regulators, accelerating flowering in *Arabidopsis thaliana*^[66,67]^. Furthermore, ABA metabolism also plays a crucial role in sweet cherry *(P. avium)* flower development and fruit setting process^[68]^. Our study revealed significant variations in the levels of eight endogenous hormones during different flower development stages, indicating their pivotal function in regulating flower development. Consistent findings have been documented in other plant species, including *Castanea henryi*^[69]^ and *Gerbera hybrida*^[70]^, further supporting the results.

Plant hormones serve as signaling molecules and can significantly impact plant growth and metabolism^[71,72]^. Our observations align with the above results, as we also detected the enrichment of DEGs in the 'plant hormone signal transduction' pathway. Moreover, compared with other hormones, the concentrations of IAA and BR showed a progressive upward trend along with flower development, so we speculate that their signaling may play critical and flowering-specific regulatory roles. Previous studies showed that auxin response factors influence leaf and flower development. It has been found that genes related to auxin biosynthesis and signal transduction contribute to the flowering of *Dendrobium officinale*^[72,73]^. Significantly, the majority of structural genes of auxin biosynthesis and signal transduction pathway, such as *AUX/IAA*, *GH3*, and *SAUR*, exhibited upregulation during the S3 stage in our investigation, highlighting their crucial involvement in stimulating flower growth. In research on flower development in sugar apples, several *Aux/IAA* genes were significantly differentially expressed, consistent with our findings^[74]^. Furthermore, in alfalfa, *MsGH3-5/6/11* acts synergistically during flower development and exhibits relatively high flower expression levels^[75]^. The *SAUR* gene family, responsible for transmitting early auxin responses, holds vital functions in plant development^[76]^. In the current study, it was evident that all DEGs affiliated with the *SAUR* gene family were specifically upregulated during the S3 stage. This observation suggests that the expression of the *SAUR* gene family potentially facilitates the developmental processes involved in flower growth. Similarly, the *SAUR* gene family could regulate pineapple's floral organ and fruit development^[77]^. Additionally, studies have shown that the *CmSAUR13*, *28*, *31*, and *39* genes exhibited pronounced expression in female flowers and played a regulatory role in the process of flower development in *Cucumis melo*^[78]^.

Plant-specific steroid hormones known as brassinosteroids (BRs) are key regulators of various physiological processes ranging from seed development to flowering and senescence^[79]^. The observed fluctuations in the expression levels of structural genes (e.g., *CYP85A1/2* and *BAK1*) associated with the brassinosteroid signaling pathway during the S3 stage suggest their potential significance in the transcriptional regulation of flower development. Notably, overexpression of the *Arabidopsis* glycosyltransferase UGT73C5, which catalyzes the glycosylation of brassinosteroids, leads to significant modifications in flower development and flowering time^[80]^. Moreover, our findings are consistent with the notion that BR signaling serves as a promoter during vegetative and reproductive development, as demonstrated in *Nelumbo nucifera*^[16]^. Furthermore, a significant association has been established between plant hormone content, as well as genes involved in plant hormone synthesis and signal transduction, with the production of floral aroma^[81,82]^. Taken together, the collective action of multiple phytohormones is crucial for the precise regulation of flower development in *T. amurensis*.

### Potential roles of genes and metabolites engaged in the terpenoid biosynthesis pathway

Plant floral fragrance is a result of volatile aromatic compounds, including terpenes, coumarins, esters, and phenolic compounds^[83]^. Terpenoids, being highly abundant in plant volatiles, serve as a primary source of floral fragrance and play a crucial role as an indicator of fruit ripeness^[84]^. We discovered that the accumulation of 20 DEMs that were annotated as terpenoids, including maslinic acid, corosolic acid, jujubogenin, and tormentic acid, increased significantly in S2 and S3, potentially being the primary cause of aroma generation in *T. amurensis* flowers. Similar results were reported in *Camellia sinensis*^[21]^ and *Vitis vinifera*^[85]^*.* In *Chrysanthemum indicum*, it was similarly demonstrated that the potential changes in floral scent are mainly caused by changes in volatile terpenoid metabolites during flower development^[86]^. According to the research conducted by Yue et al., the levels of monoterpenes present in grapes varied across their five stages of ripening, with higher concentrations observed at harvest than the initial ripening^[87]^. Furthermore, previous investigations have suggested that variations in the terpenoid components and relative terpenoid abundance were the primary factors contributing to the divergence in aroma between green and red prickly ash. These observations align with our findings in the flowers of *T. amurensis*^[84]^.

Extensive research has been conducted on the terpenoid biosynthesis pathway in various plant species, including *Jasminum sambac*^[23]^, *Camellia sinensis*^[88]^, and *Osmanthus fragrans*^[89]^. These studies have contributed to the identification of crucial genes connected to fragrance production. Nonetheless, the molecular mechanisms responsible for floral fragrance production in *T. amurensis* remain ambiguous due to the limited availability of genetic information. Remarkably, our analysis of the results unveiled a significant enrichment of DEGs in the terpenoid biosynthesis pathway, highlighting their indispensable role in the establishment of floral fragrance during development. Several key structural genes regulate the synthesis of terpenoid compounds. Recent studies identified, through comparative transcriptome analysis, *DXS*, *DXR*, *MCT*, *CMT*, and *TPS* as critical structural genes of the terpenoid biosynthetic pathway for the production of floral fragrance^[90,91]^. Within our study, a total of 165 genes associated with terpenoid biosynthesis have been successfully identified. A significant number of structural genes, such as *ACAT*, *MVK*, *TPS*, *DXS*, *DXR*, *HDS*, *HDR*, and *GPS*, exhibited considerable differential expression levels during flower development and may promote the production of floral fragrance in *T. amurensis*.

Earlier research has indicated that heightened activity of upstream genes, like *ACAT* in the MVA pathway, and *DXS* in the MEP pathway, has the potential to enhance the metabolic flux towards terpenoid biosynthesis and the production of its building blocks^[90,92]^. Our findings corroborate previous research, demonstrating that the expression profiles of *HMGS* genes in the MVA pathway were notably higher in *Lilium* 'Siberia' (known for its strong fragrance emission) compared to *Lilium* 'Novano' (known for its very faint fragrance emission)^[93]^. Specifically, in our study, we observed high expression levels of HMGS-related genes during S2, implying their potential to enhance the production of aroma compounds. Moreover, in the MVA pathway, Yan et al. reported that suppressing the *IDI* genes reduced sesquiterpene content in *Pogostemon cablin*^[94]^. The second step in the MEP pathway is facilitated by *DXR*. A study has revealed that *DXR* overexpression in transgenic tobacco increases DXR activity, increasing the levels of photosynthetic pigments and volatile isoprenoid components^[95]^. Silencing of the *HDS* and *HDR* genes in *Nicotiana benthamiana* leaves. Inhibited the MEP pathway, indicating the involvement of *HDS* and *HDR* in terpenoid biosynthesis^[96]^. Upregulation of the downstream genes *GPS* and *TPS* was observed during the flowering process. In the previous study, the biosynthesis of monoterpenes involves the production of GPP, which is carried out by *GPS*. *GPS* has been studied and characterized in multiple plant species, including *Arabidopsis*^[97]^, *Phalaenopsis bellina*^[98]^, and *Jasminum sambac*^[23]^. *TPSs* constitute a highly diversified medium-sized gene family involved in plants' terpenoid synthesis. In *Arabidopsis*, it has been documented that the biosynthesis of nearly all 20 sesquiterpenes is attributed to the enzymes *TPS21* and *TPS11*^[99]^. Furthermore, studies have revealed that the *TPS* family genes are implicated in the emission of characteristic aroma terpenes in ripe kiwifruit and *Litchi chinensis*^[100,101]^. In our investigation, we observed a significant upregulation of 13 *TPS* genes during the S3 stage, suggesting a direct association between *TPS* expression and the generation of floral fragrance. These findings coincide with the outcomes reported in the aforementioned study, reinforcing the role of *TPS* in modulating the production of floral scent.

### The functions of genes and metabolites involved in phenylpropanoid biosynthesis in floral scent production

Phenylpropanoid compounds, following terpenes, represent the second most prevalent group of floral constituents in plants. They are synthesized through a complex series of branching pathway reactions starting from phenylalanine, the initial substrate^[102]^. The phenylpropanoid synthesis pathway, also known as the cinnamic acid pathway, is a component of the phenylpropionic acid synthesis pathway^[103]^. The crucial role of the phenylpropanoid biosynthetic pathway in the production of floral fragrance has been widely studied in plants such as *Rhododendron fortune*^[103]^, *Chimonanthus praecox*^[104]^, and *Chimonanthus praecox*^[105]^. Moreover, several essential structural genes, such as *PAL*, *C4H*, *4CL*, *SAMT*, *CCOAMT*, *CCR*, *CAD*, *EGS*, and *BALDH*, are involved in regulating phenylpropanoid biosynthesis^[103]^. In the current study, KEGG enrichment analysis identified a notable enrichment of DEGs in the phenylpropanoid biosynthesis pathway, consistent with previous findings in related studies. Moreover, 74 DEGs associated with phenylpropanoid biosynthesis displayed distinct expression patterns and could potentially enhance the production of floral fragrance in *T. amurensis*. Besides structural genes, 10 metabolites of the phenylpropanoid biosynthesis pathway also showed differential accumulation, with similar results observed in *Prunus mume*^[106]^.

The *PAL* gene assumes a critical role in governing the synthesis of phenylpropanoid and benzene ring compounds. The expression profiles of *PAL*s were downregulated in the S3 stage and was significantly negatively correlated with the contents of phenylalanine, caffeic acid, and ferulic acid, which may indicate a critical negative regulatory role of *PALs* in aroma synthesis. In line with our research, a robust negative correlation was acknowledged between the downregulation of *PAL* expression and the levels of various benzene-like volatile organic compounds in *Rhododendron fortunei*, providing further support to our findings^[103]^. Using DNA microarray analysis, Verdonk and colleagues also identified that *PAL* controls the synthesis of aromatic volatile organic compounds in petunias^[107]^. Then, *4CL* can facilitate the synthesis of cinnamoyl-CoA from *t*-cinnamic acid, which in turn serves as a direct precursor for the production of benzaldehyde^[108]^. In our study, all *4CL* genes, except for *Cluster-20864.148160*, were highly expressed during the S3 stage, and many *4CL* genes were positively correlated with the contents of cinnamic acid and sinapic acid. This suggests that high expression of *4CL* may be crucial for the biosynthesis of volatile compounds responsible for the floral scent during the flowering period. Reports have indicated that two *Pm4CL* genes are significantly upregulated in *Prunus mume*, suggesting their involvement in the synthesis of volatile compounds, cinnamyl ester and eugenol^[106]^. Additionally, studies have shown that *4CL* exerts a crucial function in benzene/phenylpropane compound biosynthesis in *Ocimum sanctum*^[109]^, mulberry^[110]^, and *Agave amica*^[111]^, which aligns with our research findings. *CAD* participates in the oxidation reaction of cinnamyl alcohol to cinnamaldehyde in the phenylpropanoid metabolic pathway and is a key enzyme for aroma compound biosynthesis^[112]^. In the study, we identified the *CAD6*, *CAD10*, *CAD13*, and *CAD14* expression was positively correlated with the content of phenylpropanoid metabolites, indicating that the floral scent is affected by their expression and catalytic activities during flowering. In their extensive analysis of *Prunus mume*, Zhang et al. discovered that *PmCAD1* exerts a crucial function in the *in vitro* synthesis of cinnamyl alcohol, as evidenced by their comprehensive investigation of endogenous volatile compounds and transcriptome data^[113]^. Furthermore, transcriptomic differential expression analysis of petals from different varieties of *Rhododendron fortunei* revealed that 4 *CAD* genes were engaged in the synthesis of aromatic compounds in flowers^[103]^. Furthermore, transcriptomic differential expression analysis of petals from different varieties of *Rhododendron fortunei* revealed that four *CAD* genes were engaged in the synthesis of aromatic compounds in flowers^[114]^. Moreover, the *CCOAMT* gene expression levels in the skin of pear fruit were associated with skin color fading, which in turn affected the aroma of the pear^[115]^. These studies indicate the potential significance of the phenylpropanoid biosynthesis pathway in the development of floral scent. However, further molecular experimental investigations are needed to explore its specific mechanisms in *T. amurensis* volatile compound production in the flowers.

### TFs involved in the regulation of terpenoid and phenylpropanoid biosynthesis

The generation of compounds accountable for floral fragrance requires the expression and function of genes encoding enzymes, which are governed by regulatory genes like transcription factors^[104]^. TFs can regulate the gene expression of the biosynthetic enzymes in the floral scent synthesis pathway, thereby affecting the metabolic pathway flow toward the synthesis of specific floral scent compounds^[19]^. The first identified regulatory transcription factor of floral scent synthesis in plants is *ODO1* (*ODORANT1*) in petunias, which belongs to the *R2R3-MYB* TF family and is expressed explicitly in the petals, regulating the biosynthesis of phenylpropanoids/phenylalanine-derived compounds^[36]^. Similarly, in *Phalaenopsis orchids*, the overexpression of *bHLH4* or *bHLH6* results in elevated levels of volatile monoterpenes^[116]^. Furthermore, a recent study revealed the involvement of the *WRKY* gene in aroma synthesis through its regulation of the production of monoterpene volatiles. The expression of *OfWRKYs* is associated with monoterpene synthesis in *Osmanthus fragrans*^[117]^. So far, transcription factor family have been found to regulate terpenoid and phenylpropanoid compounds in plants, including *AP2/ERF*, *WRKY*, *NAC*, *bHLH*, *C2H2*, and *bZIP*, among others. However, there are no comprehensive reports on the transcription factors regulating floral scent synthesis in *T. amurensis*. In our investigation, we observed an overrepresentation of DEGs belonging to the *NAC*, *WRKY*, *C2H2*, *bHLH*, and *MYB* families, suggesting their potential involvement in the comprehensive regulation of flower development in *T. amurensis*. For example, *WRKY-14* the homolog of *AtWRKY71*, which has been reported to accelerate flowering by directly activating FLOWING LOCUS T and LEAFY in *Arabidopsis thaliana*, which is consistent with our findings^[118]^. Furthermore, *WRKY-21* displays significant expression during the S2 and S3 stages, exhibiting a homology with *Arabidopsis* *AtWRKY34*. This similarity suggests that *WRKY-21* likely plays a role in flowering, given that homologs in *Arabidopsis* serve as crucial controllers of pollen development^[119]^. Similarly, *MYB-6*, an orthologous counterpart of *AtMYB17*, exhibits notable expression in inflorescences and epidermal tissues, especially during the initial stages of flower development, highlighting the potential involvement of *MYB-6* in floral developmental processes^[120]^. In addition, network analysis showed significant interactions between members of different transcription factor families (such as *NAC-5*, *WRKY-14*, *bHLH-10*, and *MYB-6*) and structural genes involved in terpenoid and phenylpropanoid biosynthesis. It suggests that these TFs may regulate terpenoid and phenylpropanoid biosynthesis and accumulation, thereby affecting the production of floral fragrance. In *Citrus medica*, co-expression network analysis revealed that members of the *MYB*, *C2H2*, *NAC* TF families, and 13 *TPS* genes were associated with the synthesis of volatile terpenoid compounds^[121]^. *MYC* TFs are extensively studied in the *bHLH* family and have been shown to regulate the biosynthesis of terpenoids in numerous plant species. The *AtMYC2* transcription factor was found to positively regulate *TPS11* and *TPS21* in *Arabidopsis* flowers, resulting in an elevation of sesquiterpene emission, in agreement with our research findings^[122]^. Furthermore, we found that *C2H2-17* and *MYB-24* showed the most numerous and significant interactions with other structural genes in the terpenoid and phenylpropanoid biosynthetic pathways, respectively, and exhibited high expression levels at the S3 stage, which may indicate their central regulatory roles on the synthesis of floral fragrance. Our research findings align with results obtained in winter sweet plants, as the regulation of floral scent compound biosynthesis in winter sweet involves the differential expression of 56 *C2H2* transcription factors^[104]^. In addition, in *Arabidopsis*, the *C2H2* TF family member *AtZAT10* was shown to regulate the expression of ABA signaling pathway genes, indirectly affecting the synthesis of terpenoid compounds. Furthermore, four *MYB* members regulate phenylpropanoid volatiles in petunias^[123]^, overexpression of *Arabidopsis* *MYB* genes in petunia flowers demonstrated that regulating the flow of phenylpropanoid precursor metabolism affects floral scent synthesis^[124]^. Moreover, overexpression of the *R2R3-MYB* transcription factor *PtMYB14* from *Pinus taeda* leads to the accumulation of sesquiterpenes in transgenic white spruce (*Picea glauca*) plants^[34]^. The *PmMYB4* was shown to regulate downstream structural genes, leading to significant differences in aroma compounds among different cultivars, which is consistent with our research findings^[106]^. The findings above suggest that TFs assume a crucial role in regulating terpenoid and phenylpropanoid biosynthesis through the modulation of downstream structural genes, ultimately modulating floral scent production.

## Conclusions

**Figure 10:**
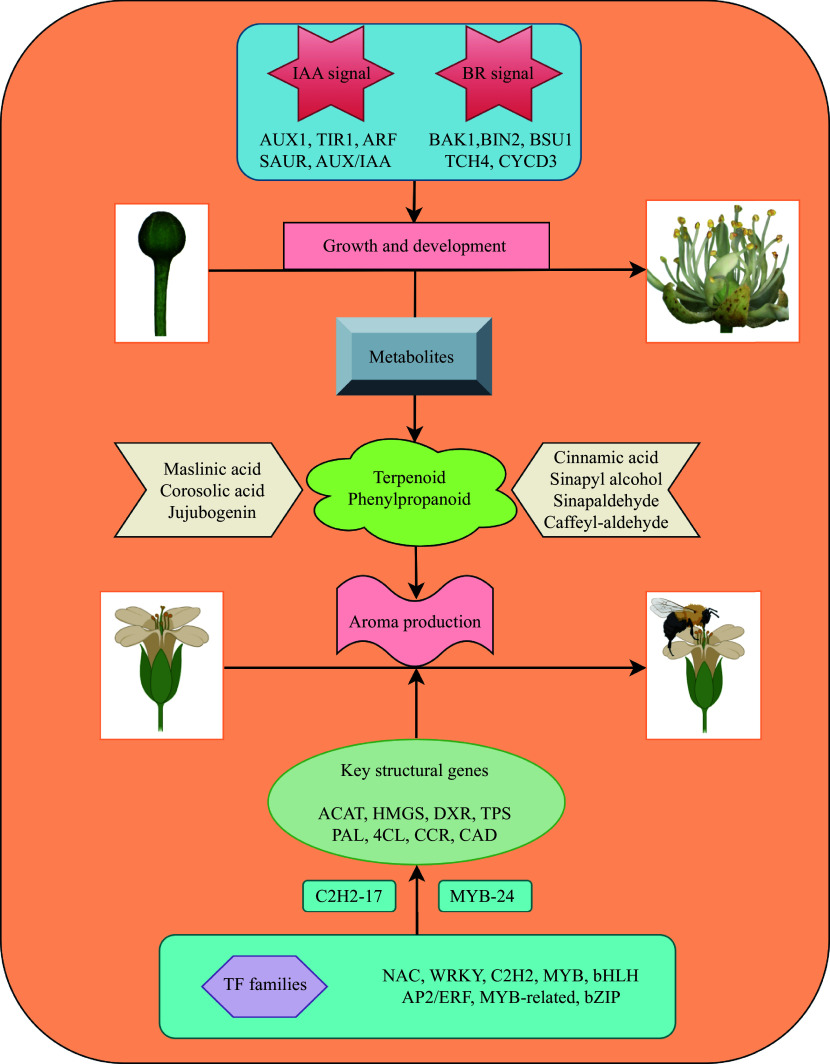
Diagram illustrating the process of flower development and aroma synthesis in the nectar tree (*Tilia amurensis*).

In summary, we utilized metabolomics to quantify and characterize the secondary metabolites accumulated during the development of *T. amurensis* flowers. We further conducted a transcriptomic analysis of the flowers during different flowering stages. A total of 89,526 DEGs and 664 DEMs were discovered in the current study, and enrichment analysis indicated that the biosynthesis pathways of terpenoids and phenylpropanoids were commonly enriched in the different comparison groups. Moreover, the roles of two key plant hormones, IAA and BR, in flower development were evaluated based on their accumulation and the expression patterns of their signal transduction and biosynthesis pathways. Through the integration of transcriptomics and metabolomics techniques, we successfully determined the temporal accumulation patterns of essential metabolites, such as maslinic acid, jujubogenin, cinnamic acid, and sinapic acid. We identified that terpenoids and phenylpropanoids are the major metabolites involved in the production of floral fragrances. We also identified 165 and 74 DEGs associated with terpenoid and phenylpropanoid biosynthesis, respectively, and further predicted the underlying regulatory mechanisms involving structural genes such as *ACAT*, *HDS*, *TPS*, *4CL*, *CAD*, and *CCOAMT* in the synthesis of both compound classes. Furthermore, in this study, we uncovered the involvement of transcription factor families such as *NAC*, *WRKY*, and *MYB* in regulating terpenoid and phenylpropanoid biosynthesis pathways in *T. amurensis* flowers. We identified the transcription factors *C2H2-17* and *MYB-24* as central regulators with positive regulatory roles in the terpenoid and phenylpropanoid biosynthesis pathways, respectively. These findings lay the foundation for the generation of floral fragrance in *T. amurensis* (Fig. 10).

## SUPPLEMENTARY DATA

Supplementary data to this article can be found online.

## Data Availability

RNA-Seq raw data from three samples were deposited in the National Center for Biotechnology Information (NCBI) under the accession number PRJNA962507.
