# Supplementary material for: Deciphering aroma formation during flowering in nectar tree (Tilia amurensis): insights from integrated metabolome and transcriptome analysis
Source: For Res (Fayettev). 2023 Oct 8;3:24. doi: 10.48130/FR-2023-0024 (PMC11524258; doi:10.48130/FR-2023-0024)
Supplement: Supplementary file 1 — Supplementary data to this article can be found online. [file FR-2023-0024-S1.zip › 10.48130_FR-2023-0024-Suppl-FigureS1.pdf]

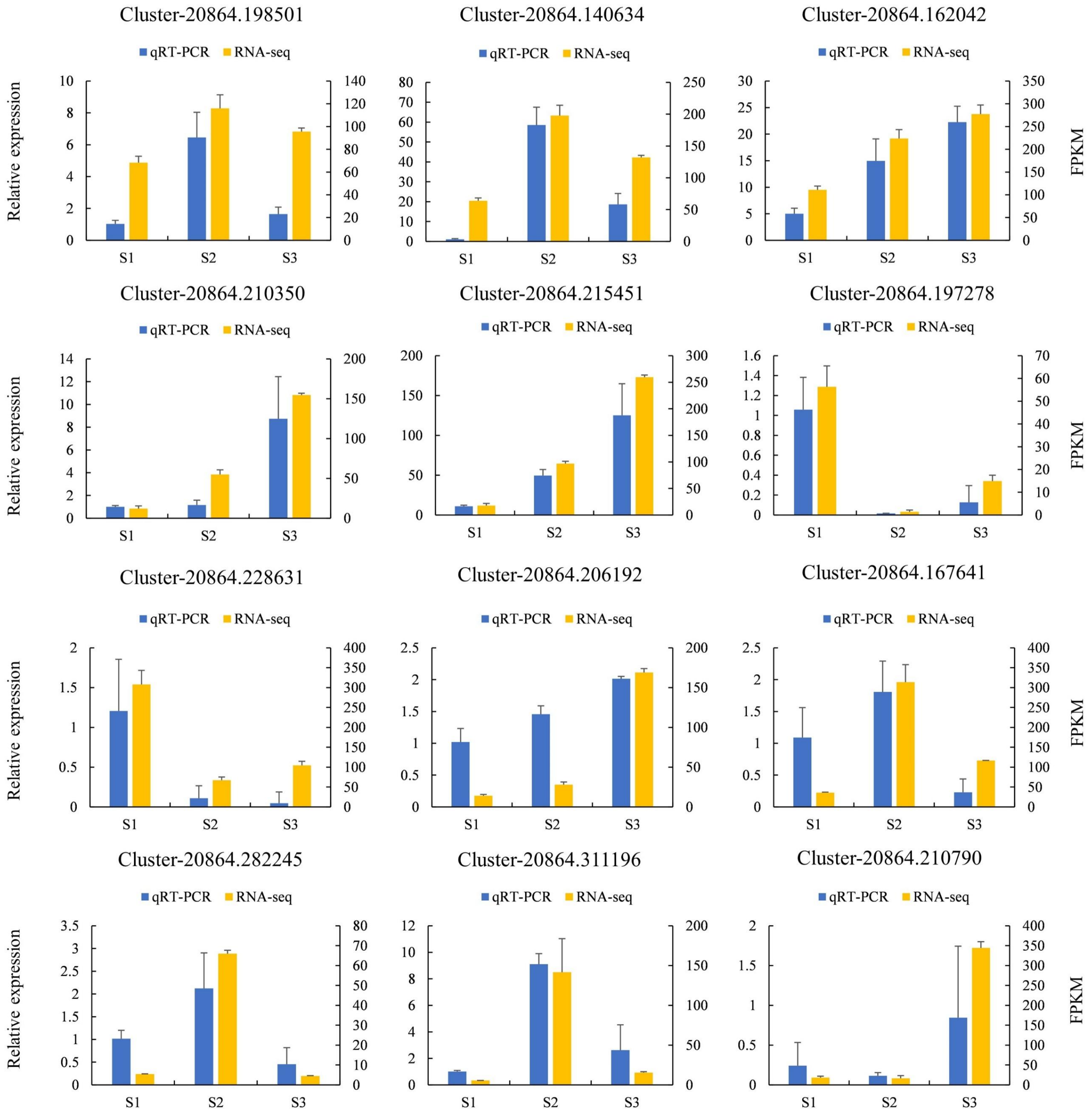

Supplemental Figure 1. Gene expression patterns of RNA-seq and qPCR. The X-axis represents three samples (S1, S2 and S3). The Y-axis on the left represents the expression data of qRT-PCR. The Y-axis on the right represents the relative expression levels of DEGs validated by RNA-seq. The error bars represent standard error.
