# Supplementary material for: Deciphering aroma formation during flowering in nectar tree (Tilia amurensis): insights from integrated metabolome and transcriptome analysis
Source: For Res (Fayettev). 2023 Oct 8;3:24. doi: 10.48130/FR-2023-0024 (PMC11524258; doi:10.48130/FR-2023-0024)
Supplement: Supplementary file 1 — Supplementary data to this article can be found online. [file FR-2023-0024-S1.zip › 10.48130_FR-2023-0024-Suppl-FigureS3.pdf]

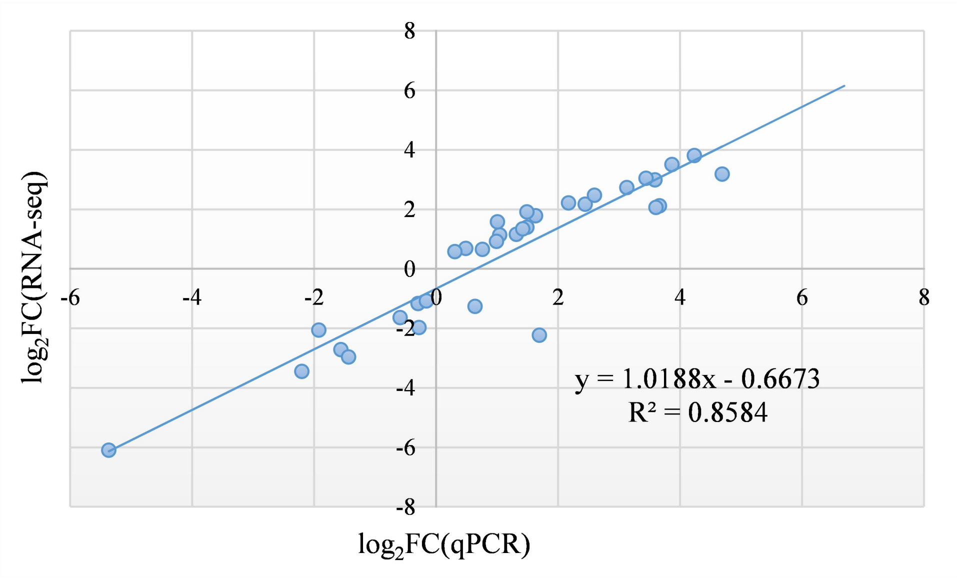

Supplemental Figure 3. The consistency of RNA-seq and qPCR data was demonstrated based on scatter plot. X-axis represents $\log_2$(RNA-seq), Y-axis represents $\log_2$(qPCR).
